# Improving Interference Control in ADHD Patients with Transcranial Direct Current Stimulation (tDCS)

**DOI:** 10.3389/fncel.2016.00072

**Published:** 2016-03-22

**Authors:** Carolin Breitling, Tino Zaehle, Moritz Dannhauer, Björn Bonath, Jana Tegelbeckers, Hans-Henning Flechtner, Kerstin Krauel

**Affiliations:** ^1^Department of Child and Adolescent Psychiatry and Psychotherapy, University of MagdeburgMagdeburg, Germany; ^2^Department of Neurology, University of MagdeburgMagdeburg, Germany; ^3^Scientific Computing and Imaging Institute, Center for Integrated Biomedical Computing, University of UtahSalt Lake City, UT, USA; ^4^Center for Behavioral Brain Sciences, University of MagdeburgMagdeburg, Germany

**Keywords:** transcranial direct current stimulation, tDCS, attention deficit hyperactivity disorder, ADHD, interference control, inhibitory control, right inferior frontal gyrus, flanker task

## Abstract

The use of transcranial direct current stimulation (tDCS) in patients with attention deficit hyperactivity disorder (ADHD) has been suggested as a promising alternative to psychopharmacological treatment approaches due to its local and network effects on brain activation. In the current study, we investigated the impact of tDCS over the right inferior frontal gyrus (rIFG) on interference control in 21 male adolescents with ADHD and 21 age matched healthy controls aged 13–17 years, who underwent three separate sessions of tDCS (anodal, cathodal, and sham) while completing a Flanker task. Even though anodal stimulation appeared to diminish commission errors in the ADHD group, the overall analysis revealed no significant effect of tDCS. Since participants showed a considerable learning effect from the first to the second session, performance in the first session was separately analyzed. ADHD patients receiving sham stimulation in the first session showed impaired interference control compared to healthy control participants whereas ADHD patients who were exposed to anodal stimulation, showed comparable performance levels (commission errors, reaction time variability) to the control group. These results suggest that anodal tDCS of the right inferior frontal gyrus could improve interference control in patients with ADHD.

## Introduction

Attention deficit hyperactivity disorder (ADHD) is a childhood-onset psychiatric disorder which is characterized by developmentally inappropriate levels of inattention, impulsivity and hyperactivity (DSM-IV). It has a worldwide prevalence of 5.3% in children (Polanczyk et al., 2007) and in many cases symptoms persist into adulthood (Mannuzza et al., 2003) with an adult prevalence of still 3.4% (Fayyad et al., 2007).

In ADHD cognitive control, the ability to control sensory processes and actions in a goal-directed manner (Bunge et al., 2002), is severely compromised, affecting motor, emotional and cognitive domains (Wodka et al., 2007). ADHD patients are particularly impaired in different aspects of inhibition control, namely interference control, the suppression of task irrelevant, competing stimuli and response inhibition, the suppression of a prepotent response. Interference control has been effectively investigated using the Flanker task (Eriksen and Eriksen, 1974) and the Simon task (Simon, 1990), which has been shown to provide a reliable measurement of this cognitive ability (Wöstmann et al., 2013). In both tasks participants have to indicate the category of a target stimulus by a right or a left button press. In the Flanker task, the target is surrounded by distracting stimuli which must be ignored in order to give the right response. In the Simon task, the target stimulus is presented either on the left or on the right side. In this case, the position must be ignored to give the right response. So far, ADHD patients have shown higher error rates and slower reaction times compared to healthy controls (Mullane et al., 2009).

Successful interference control has been associated with the integrity of the right inferior frontal gyrus (rIFG) (Luks et al., 2010; Zhu et al., 2010). In children and adolescents with ADHD various studies have revealed structural (Sowell et al., 2003; Durston et al., 2004) as well as functional alterations (Aron and Poldrack, 2005) in the rIFG. For example during a Simon task, unmedicated ADHD patients showed less activity in the rIFG compared to healthy controls (Rubia et al., 2011) whereas ADHD patients medicated with methylphenidate did not differ from healthy controls (Lee et al., 2010). Thus, increasing activity of the rIFG seems to facilitate interference control.

Transcranial direct current stimulation (tDCS) is a non-invasive tool for modulating cortical excitability. To conduct tDCS a weak current is passing through the scalp mostly via two conductive rubber electrodes in sponges soaked in saline solution or covered with conductive gel. The modulation of cortical excitability depends on the polarity of electrodes. In general, the positively charged anode increases cortical excitability while the negatively charged cathode decreases it (Nitsche and Paulus, 2000). This modulation is due to a modification of the resting membrane potential in regions of current flow (Stagg and Nitsche, 2011). TDCS when applied for 30 min induces prolonged effects after the end of stimulation (Nitsche and Paulus, 2001).

Studies showed tDCS induced improvements of symptom severity already in different psychiatric and neurologic disorders, for example depression (Kalu et al., 2012), schizophrenia (Brunelin et al., 2012), stroke (Chang et al., 2015) and dyslexia (Heth and Lavidor, 2015). Castellanos and Proal (2012) suggested that tDCS may also be of therapeutic use for ADHD, especially due to its beneficial effect on larger scale networks (Keeser et al., 2011). The development of non-pharmaceutical treatment approaches is particularly relevant in ADHD, since even though many patients benefit from medical treatment, a substantial number report remarkable side effects and parents as well as children and adolescents often wish for alternative treatment strategies (Halperin and Healey, 2011). Effects of stimulant treatment persist only for the time of active medication (Chronis et al., 2003), whereas beneficial effects of repetitive tDCS have been reported to last for several month (Cohen Kadosh et al., 2010). Even though tDCS has been predominantly employed in adults, studies in children and adolescents have confirmed that this method is also well tolerated and save in younger age groups (Mattai et al., 2011; Andrade et al., 2014; Moliadze et al., 2014; Krishnan et al., 2015).

Studies testing modulatory effects of tDCS in ADHD are, however, sparse. Using oscillatory tDCS during slow wave sleep, Prehn-Kristensen et al. (2014) demonstrated an improvement of declarative memory performance on the next day as well as improved reaction times in a go/nogo task in children with ADHD (Munz et al., 2015).

In the current study, we aimed to improve interference control in adolescents with ADHD using tDCS. In healthy adults, tDCS over of the rIFG and the pre-supplementary motor area has been successfully used to improve response inhibition (Hsu et al., 2011; Jacobson et al., 2011; Ditye et al., 2012), thus, the rIFG was chosen as target region for stimulation in adolescents with and without ADHD. We applied anodal, cathodal, and sham tDCS to each participant. We predicted that ADHD patients would show impaired interference control in the sham condition and would improve through anodal tDCS over the right IFG. Since cathodal tDCS over frontal cortical areas can impair as well as facilitate cognitive processes (Nozari et al., 2014), we included cathodal stimulation for explorative purposes. Finally, we performed computer simulations using a pediatric model in order to model the current flow in the experimental stimulation design.

## Materials and Methods

### Participants

Forty six male adolescents aged 13–17 years participated in the study. Three ADHD patients and one control participant were not included because behavioral data suggested that they did not comprehend the task (see Statistics for exclusion criteria).

Participants and their parents were interviewed with the Revised Schedule for Affective Disorders and Schizophrenia for School-Age Children: Present and Lifetime Version (K-SADS-PL, Kaufmann et al., 1997). Twenty one of the adolescents met the diagnostic criteria of the DSM-IV for ADHD (16 combined subtype, 5 primarily inattentive subtype). Additionally, one patient fulfilled diagnostic criteria for conduct disorder. Control subjects had no history of neurological or psychiatric disorders.

Intelligence was assessed by the Culture Fair Test – Revised Version (CFT, Weiss, 2008). Handedness was assessed by the Edinburgh Handedness Inventory (Oldfield, 1971). **Table 1** shows that the groups did not differ in age or intelligence. Parental and self report revealed that ADHD symptom severity was higher in ADHD patients compared to healthy controls. Two participants of the ADHD group and two participants of the control group were left handed, all others were right handed. ADHD patients taking methylphenidate (*n* = 10, sustained-release) or lisdexamfetamine (*n* = 1) for the treatment of ADHD, refrained at least 24 h before each experimental session from taking their medication.

**Table 1:** Group characteristics.

	ADHD (*n* = 21)	Controls (*n* = 21)	*p*-value
Age (years)	14.33	14.24	0.79
Combined subtype (ADHD)	16	–	–
Primarily inattentive subtype (ADHD)	5	–	–
Conduct disorder	1	–	–
Medication	11	–	–
IQ (CFT)	100.00	105.33	0.14
ADHD symptom severity (K-SADS-PL, parental rating present)	10.86	0.25	<0.001
ADHD symptom severity (K-SADS-PL, self rating present)	9.90	0.10	<0.001

No participant reported contraindications to receiving tDCS. The study was approved by the local ethics committee of the University of Magdeburg and followed the ethical standards of the Helsinki declaration. All participants and their parents gave written informed assent/consent before participating. Each participant received a voucher (10€) for a local shopping center per session.

### Experimental Design and Task

**FIGURE 1:**
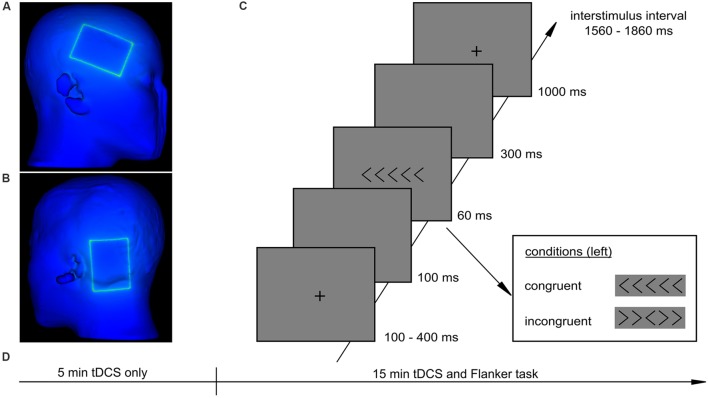
**Experimental procedure and task. (A)** position of the stimulation electrode, **(B)** position of the reference electrode, **(C)** Flanker task, **(D)** procedure of an experimental session.

Each participant received anodal, cathodal, and sham tDCS, separated by at least 1 week, while completing a modified Eriksen Flanker task (Eriksen and Eriksen, 1974). The order of tDCS types was pseudo-randomized and counterbalanced and participants were blinded to the type of stimulation they received in each session. For the Flanker task, stimuli consisted of one central target arrow flanked by two arrows on both sides (**Figure 1C**). Participants had to indicate the direction of the target arrow (right/left) by a button press with the index finger of their right or left hand, respectively. Flanking arrows pointed in the same (congruent stimuli) or opposite direction (incongruent stimuli) as the target arrow. Four resulting arrays were randomly presented with equal frequency. Stimuli were presented with a visual angle of 3.8° with Presentation software (version 16.4, www.neurobs.com).

As illustrated in **Figure 1**, each trial started with a fixation cross, presented for 100, 200, 300, or 400 ms followed by a blank screen for 100 ms. Then stimuli were presented for 60 ms followed by a blank screen for 300 ms and a fixation cross for 1000 ms. Participants had a total time of 1360 ms to respond to the target stimulus. The interstimulus interval varied between 1560 and 1860 ms. Participants were instructed to react as accurately and as fast as possible. The task consisted of three runs à 156 trials and had a total duration of 15 min. Before the task started, a short training was conducted for 1 min (32 trials).

### Transcranial Direct Current Stimulation

A battery driven DC stimulator (neuroConn, Germany) delivered a direct current with an intensity of 1 mA via two conductive rubber electrodes (7 × 5 cm, current density: 0.029 mA/cm^2^) covered with saline soaked sponges. The stimulation electrode was centered on electrode site F8 (**Figure 1A**), according to the 10–20 International EEG system which corresponds to the rIFG (Homan et al., 1987). The reference electrode was placed posterior to the left mastoid (**Figure 1B**). Anodal and cathodal tDCS were applied for 20 min (with a 30 s ramp up and down). For sham stimulation electrode arrangement was identical to active stimulation but the stimulator was turned off after 30 s with a 30 s ramp up and down. To ensure stable stimulation effects according to Nitsche et al. (2008), each participant received 5 min of tDCS before he started with the Flanker task (**Figure 1D**). In the first 5 min of stimulation instruction was given to the participants and a short training block was conducted. Afterward, stimulation continued for the 15 min duration of the experimental task. At the end of each session participants indicated the strength of skin sensations caused by tDCS and their subjective ability to concentrate on a 5-point Likert-scale.

### Computer Simulation of tDCS

The simulations were conducted using a head model, which was derived from a pediatric brain atlas covering subjects in an age range of 9–18 years. This multimodal head model [Pediatric Head Modeling (PHM); Song et al., 2013, https://home.pedeheadmod.net/display/Pedvol/Pediatric+Head+Atlasses#, accessed on May 19th 2015] fuses CT imaging data of a 13-year-old boy, which is registered non-linearly, with an MRI atlas (MNI, Fonov et al., 2011). The multimodal approach combines advantages of computed tomography (CT) and magnetic resonance imaging (MRI) for an accurate representation of tissues, such as scalp, skull, internal air (CT) as well as gray/white matter and eyeballs (MRI). The segmentation (PHM) was utilized to create a tetrahedral mesh (software: cleaver version 1.5.4, 6.7/38.3 million tetrahedral nodes/elements), which was used later on to perform bioelectric simulations of tDCS (finite element method, Dannhauer et al., 2012). Two electrodes sponges (50 × 70 mm, 5 mm thickness) were meshed with the head model based on experimental electrode positioning (anode: F8, cathode: P7). SCIRun5 (SCI-Institute, 2016) was employed to set up electrical properties such as boundary conditions (±1 mA, complete electrode model), for anode and cathode, as well as, isotropic conductivities (scalp = 0.43, skull = 0.01, CSF = 1.79, gray and white matter = 0.33/0.142, eyeballs = 0.4, electrode saline = 1.4 and internal air = 1e-6 [S/m]) and electrode contact impedance of 20 kΩ. Again, SCIRun5/BrainStimulator was applied to further compute a finite element solution and visualize current densities.

### Statistics

From the original sample, three ADHD patients and one control person were excluded from the analysis because their commission error rates were more than two standard deviations above the mean of their respective group (all stimuli, first session) indicating that they did not entirely comprehend the task.

Rates of commission errors (false button press) and omission errors (no button press), mean of reaction times and reaction time variability (standard deviation of reaction time normalized by mean of reaction time) were analyzed in SPSS (version 22.0). Incongruent trials were considered for all analyses, as the performance in these trials serves as an indicator for quality of interference control. Only the first reaction of participants was analyzed and only correct trials were considered for reaction time analyses. Trials with reaction times less than 200 ms were excluded from all analyses. Repeated measures analyses of variance (ANOVA) were conducted with the factors tDCS type (anodal vs. cathodal vs. sham) and group (ADHD vs. control) for all dependent measures. In order to control for learning effects across sessions, we separately analyzed session number (1 vs. 2 vs. 3) and group (ADHD vs. control) in a further repeated measures ANOVA. Mean comparisons of directed hypotheses are reported one-sided.

## Results

**FIGURE 2:**
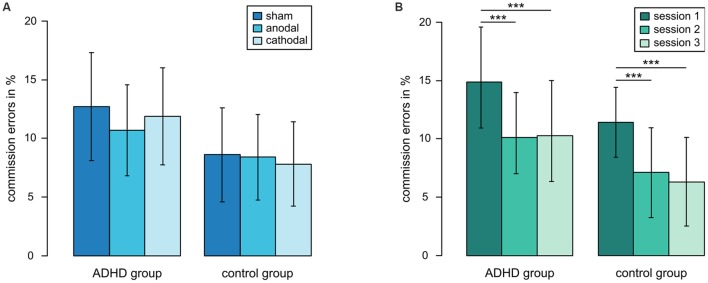
**Commission errors in Flanker task. (A)** commission errors in different tDCS conditions when three sessions of each participant are considered, **(B)** learning effect, ***p < 0.001, error bars represent one standard deviation.

The overall ANOVA did not show effects of tDCS regarding commission errors, omission errors, reaction times and reaction time variability, although descriptive data suggested diminished commission errors in the ADHD group after anodal stimulation (**Figure 2A**, Supplementary Table S1). The analysis of session number, however, revealed a significant learning effect for commission errors [*F*_(2,80)_ = 15.71, *p* < 0.001], reaction times [*F*_(2,80)_= 6.81, *p* < 0.01] and reaction time variability [*F*_(2,80)_= 3.88, *p* < 0.05]. Participants of both groups made more errors in the first session compared to the second [*t*_(41)_= 4.63, *p* < 0.001] and the third session [*t*_(41)_= 4.38, *p* < 0.001] (**Figure 2B**). Reaction times and reaction time variability decreased also from the first to the second session [*t*_(41)_= 2.99, *p* < 0.01, *t*_(41)_= 2.81, *p* < 0.01]. See Supplementary Table S2 for detailed values. To resolve the confounding of learning and stimulation effects, we subsequently focused in an exploratory analysis on the first session of each participant. The three resulting ADHD groups (anodal, cathodal, sham, *n* = 7 in each group) as well as the three control groups did not differ in age or intelligence. Symptom severity did not differ between ADHD groups and the distribution of clinical subtypes and medication was comparable (all *p* > 0.1).

**FIGURE 3:**
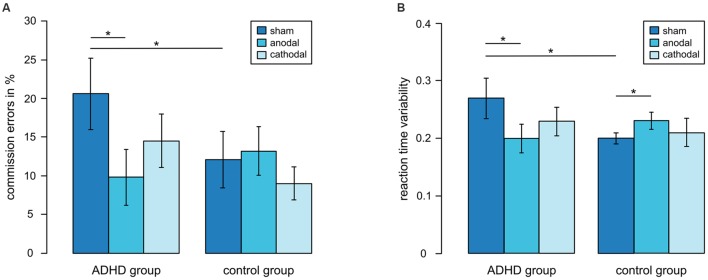
**Performance for first sessions in different tDCS conditions. (A)** Commission errors, **(B)** reaction time variability, *p < 0.05, error bars represent one standard deviation.

A marginally significant interaction between tDCS stimulation and group showed that patients tended to respond more strongly to stimulation than controls [*F*_(2,36)_ = 2.71, *p* = 0.08] (**Figure 3A**). ADHD patients who received sham stimulation made more commission errors (mean: 20.57%) than controls (mean: 12.08%) [*t*_(12)_ = -1.92, *p* < 0.05] and ADHD patients receiving anodal stimulation had lower commission error rates (mean: 9.82%) than those who received sham stimulation [*t*_(12)_ = -2.44, *p* = 0.02]. In the control group, there was no effect of tDCS (anodal: 13.21%, sham: 12.08%). Thus, the commission error rate in ADHD patients who received anodal tDCS was at the same level as in healthy controls. Cathodal tDCS did not differ from sham tDCS in both groups (ADHD: 14.45%, controls: 8.97%).

The same pattern occurred for reaction time variability (**Figure 3B**). An interaction between tDCS stimulation and group [*F*_(2,36)_ = 3.47, *p* < 0.05] showed that in patients reaction time variability was reduced during anodal stimulation compared to sham stimulation [*t*_(12)_ = -2.02, *p* < 0.05]. Whereas ADHD patients who received sham stimulation showed higher variability of reaction times than control participants [*t*_(7.7)_ = -2.66, *p* = 0.01], ADHD patients in the anodal condition did not differ from the control group. In the control group, reaction time variability was lower during sham tDCS than during anodal tDCS [*t*_(12)_ = 2.03, *p* < 0.05]. Cathodal tDCS did not differ from sham tDCS in both groups.

For omission errors and means of reaction times there were no significant main effects of tDCS type or group and no interaction between both factors (see Supplementary Table S3).

### Side Effects

Participants judged their ability to concentrate similarly for all different tDCS types. For skin sensations, there was a trend toward a main effect of tDCS [*F*_(2,36)_ = 2.83, *p* = 0.07]. Skin sensations (**Table 2**) were rated higher during cathodal tDCS compared to sham tDCS [*t*_(26)_ = 2.07, *p* < 0.05].

**Table 2:** Skin sensations and perceived concentration during tDCS sessions (mean and standard deviation).

	Sham	Anodal	Cathodal	Main effect tDCS
Skin sensations	2.14 (1.10)	2.43 (0.85)	3.07 (1.27)	[*F*_(2,36)_ = 2.83, *p* = 0.07], c > s
Concentration	4.36 (0.75)	3.93 (0.62)	4.07 (0.73)	[*F*_(2,36)_ = 1.36, *p* = 0.27]

*1 = “not,” 2 = “slightly,” 3 = “fairly,” 4 = “quite,” 5 = “very.”*

### Computer Simulation of tDCS

**FIGURE 4:**
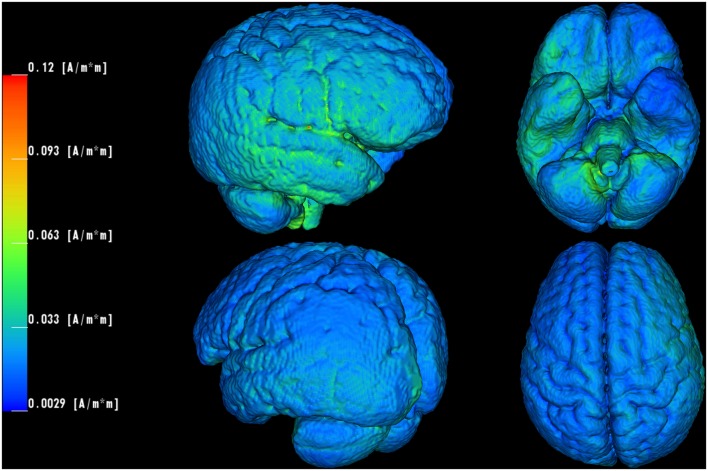
**Computer simulation of the utilized tDCS electrode settings.** Current density concentrations are visualized on brain surface of the employed pediatric head model. The current density values are peaking locally in frontal/temporal (maximum: 0.12 A/m^2^), brain stem and lower cerebellar (near foramen magnum) regions.

**Figure 4** shows that the computer model predicted current density concentrations over right frontal/temporal regions. In more detail, the anodal current flew through scalp tissue, while a fraction entered skull/CSF to reach brain tissue at right frontal/temporal areas. The stimulation estimated higher current density concentrations at brain stem/lower cerebellum close to the foramen magnum. Some amount of that current might have left the cranium through the foramen magnum, as indicated in Eichelbaum et al. (2014), flowing further toward the cathodal electrode.

## Discussion

The aim of this study was to demonstrate the impact of tDCS over the rIFG on interference control in ADHD patients. Accordingly, adolescents with and without ADHD underwent anodal, cathodal and sham tDCS while completing a Flanker task. In contrast to our prediction, the overall analysis did not reveal a significant tDCS effect in either group. However, a significant learning effect from the first to the second session suggested that effects of learning and stimulation could have overlapped or interacted. When only the first session of each participant was analyzed, ADHD patients receiving anodal tDCS showed significantly lower commission error rates and reaction time variability than patients who obtained sham stimulation. Moreover, task performance in the anodal ADHD group did not differ from the healthy control group whereas performance in the sham ADHD group did. It is conceivable that while errors were diminished, reaction times were not increased, indicating that anodal stimulation was not associated with a speed accuracy trade off. Cathodal tDCS did not influence performance of participants. It is worth noting that significant results in the overall analysis were most likely made more difficult by the considerably large variability in performance and tDCS effects between participants. Different studies have recently shown (Kim et al., 2014; Lopez-Alonso et al., 2014; Wiethoff et al., 2014) that not all individuals profit from tDCS and it is highly debated to what extent individual differences in anatomy, neurophysiology, neurochemistry, genetics or psychological status can predict responders and non-responders (Li et al., 2015).

Analyzing the first session, error rate considerably differed, by approximately 50%, between the sham and the anodal ADHD group equaling the extent of error reduction in a Flanker task by methylphenidate (Jonkman et al., 2007). Contrary to our expectations, we found tDCS effects only in the first session. This result is consistent with studies of the motor cortex showing that stimulation effects in second tDCS sessions were diminished and delayed (Monte-Silva et al., 2010) or even reversed (Monte-Silva et al., 2013) suggesting that anodal tDCS caused a slight inhibition. Gill et al. (2015) found anodal tDCS effects when stimulating during high but not during low working memory load. In contrast, Bortoletto et al. (2015) found tDCS effects only in an easy task condition, but not in a difficult one. These results indicate that the current state of the stimulated area depending on numerous factors can strongly modulate stimulation effects. In our study the first session was probably associated with high levels of arousal and participants had no experience with the task. These factors may have set cortical activation of ADHD patients into a state properly for tDCS to interact with. Alternatively, learning effects could have been enhanced by stimulation (Ditye et al., 2012), which would lead to better than expected task performance in subsequent sham sessions (see Supplementary Tables S4 and S5).

In contrast to existing studies which achieved improvements in a broad range of cognitive abilities in healthy persons (Coffman et al., 2014) we did not find any improvements in the control group. It could be speculated that in deficient interference control an enhancement of the rIFG activity will result in an improvement of this cognitive process, whereas in an optimal level of cognitive processing – as in our sample of healthy control subjects – additional modulation will be less effective or even detrimental. This might be related to an inverted U-shaped dose-response relationship between cortical activity and cognitive outcome, as it has been reported for the relation of the dose of a pharmacological treatment and altered cognitive functions (Goldman-Rakic et al., 2000). Monte-Silva et al. (2009) demonstrated dose-dependent impairment by a dopamine D2-like agonist on anodal tDCS-induced motor cortex excitability. The findings revealed an inverted U-shaped curve with enhanced activity by anodal tDCS at an optimal dose of D2-like agonists, whereas lower and higher doses resulted in less activity (Monte-Silva et al., 2009; Krause et al., 2013).

We assume that improvement of interference control is caused by an enhanced activity of the rIFG. The computer simulation supports our experimental results and suggests that we succeeded in targeting this area. However, since we did not exclusively stimulate the rIFG, effects could also be based on other physiological mechanisms. Using large electrodes (surface of 35 cm^2^) produced widespread changes in cortical excitability. This might have led to changes in the general arousal (McIntire et al., 2014) or could have influenced cortical connectivity (Pena-Gomez et al., 2012). It is unlikely, however, that unspecific tDCS effects independent of electrode montage are responsible for those effects since there was no significant improvement with respect to the cathodal tDCS condition. Small current density values, predicted on brain and scalp model surface, reflect the fact that shunting through low resistive head tissues (e.g., skull) diminish largely when injecting through two big remote patch electrodes.

### Limitations

One limitation of our study is the small sample size in our exploratory analysis of the first session. A small sample size bears the risk that confounding variables are not equally distributed between groups and could therefore add to the experimental effect. To control for confounding variables, participants were assigned randomly to experimental conditions. Moreover, experimental groups of this study did not differ in age, intelligence, ADHD symptom severity and ADHD subtypes. Nevertheless, it cannot be stated with certainty that randomization was successful for all possible confounding variables. Therefore, to assess genuine pre-stimulation differences between groups, a stimulation naïve session should be included in future investigations.

Since simple learning effects can mask effects of stimulation, the use of tasks with the prospects of small learning or practice effects would be essential. Commission error rates in the flanker task have been actually shown to provide satisfactory test–retest reliability (Wöstmann et al., 2013). Alternatively, established neuropsychological tests with parallel forms could provide useful estimates of performance increments due to stimulation.

Between participants, there was a considerable variability of error rates, especially in ADHD patients. ADHD symptoms improve with ongoing age (Faraone et al., 2006) but this development proceeds differently in each individual. Thus, a younger sample would possibly have shown a more homogenous behavior which could increase the likelihood that positive effects of anodal stimulation are more clearly detectable.

Currently, the view that sham stimulation is an appropriate method for blinding participants is questioned (Davis et al., 2013; Horvath et al., 2014). As a contribution to this discussion we found that skin sensations were not identical between sham and cathodal tDCS. In adults, it was already shown that sham tDCS is not an appropriate blinding method when using higher current intensities of 2 mA (O’Connell et al., 2012). It seems that for adolescents blinding is problematic even at a current intensity of 1 mA, as skin sensitivity is higher during younger age (Lautenbacher and Strian, 1991). This outcome should find attention when conducting and interpreting results of tDCS studies with children and adolescents.

### Future Directions

Transcranial direct current stimulation studies with young participants have reported only mild and transient side effects, like tingling and itching (Krishnan et al., 2015) and the method is considered save for children and adolescents when safety guidelines are followed. On this basis, tDCS is a promising tool for the treatment of childhood onset psychiatric disorders, since it provides the particular opportunity to positively influence atypical brain development early and persistently (Krause and Cohen Kadosh, 2013). At the same time, it is important to bear in mind that tDCS can also cause deterioration of cognitive functions (Sellers et al., 2015).

Our results suggest that the used tDCS assembly could be suitable for improving interference control in ADHD patients. In further investigations results have to be confirmed and extended. For example, it needs to be clarified whether targeting specific functions such as interference control via tDCS leads to amelioration of clinical symptoms such as general behavioral control or impulsivity. For the development of an effective therapy it will be essential to investigate how long-term effects can be accomplished and maintained. In this context, it is important to further investigate potential learning or carry-over effects, since clinical treatment efficacy is often assessed in cross-over designs which could underestimate effects of stimulation.

Finally, anatomic inter-subject variability is a particular challenge for the development of stimulation protocols because factors like the topography of the cortex, subcutaneous fat and distribution of cerebrospinal fluid determine current flow (de Berker et al., 2013) and can lead to different activations in different individuals (Horvath et al., 2014). Perspectively, computer simulations could aid to identify electrode settings that combine behavioral improvement with minimal dosage (in terms of stimulation intensity as well as spread of current within the brain), which is particularly important in pediatric populations.

## Author Contributions

All authors listed, have made substantial, direct and intellectual contribution to the work, and approved it for publication.

## Conflict of Interest Statement

The authors declare that the research was conducted in the absence of any commercial or financial relationships that could be construed as a potential conflict of interest.
