# Supplementary material for: Improving Interference Control in ADHD Patients with Transcranial Direct Current Stimulation (tDCS)
Source: Front Cell Neurosci. 2016 Mar 22;10:72. doi: 10.3389/fncel.2016.00072 (PMC4834583; doi:10.3389/fncel.2016.00072)
Supplement: Supplementary file 1 [file Supplementary_Material_Breitling.PDF]

Supplementary Material

Improving interference control in ADHD patients with transcranial direct current stimulation (tDCS)

Carolin Breitling*, Tino Zaehle, Moritz Dannhauer, Björn Bonath, Jana Tegelbeckers, Hans-Henning Flechtner, Kerstin Krauel

*** Correspondence:** Carolin Breitling: carolin.breitling@med.ovgu.de

Supplementary Table 1. Means, standard deviations and results of ANOVA in all sessions for sham, anodal and cathodal tDCS.

	ADHD sham	ADHD anodal	ADHD cathodal	Control sham	Control anodal	Control cathodal	Main effect tDCS	Main effect group	Interaction tDCS x group
Commission errors	12.7% (9.2%)	10.7% (7.8%)	11.9% (8.3%)	8.6% (8.0%)	8.4% (7.3%)	7.8% (7.1%)	$F_{(2,80)} = 0.49$	$F_{(1,40)} = 2.80$	$F_{(2,80)} = 0.41$
Omission errors	4.1% (8.9%)	4.5% (9.2%)	7.1% (11.6%)	2.0% (6.8%)	0.9% (2.0%)	1.9% (4.7%)	$F_{(2,80)} = 2.29$	$F_{(1,40)} = 2.65$	$F_{(2,80)} = 1.61$
Reaction time	567 ms (46 ms)	573 ms (52 ms)	560 ms (46 ms)	544 ms (81 ms)	550 ms (85 ms)	544 ms (79 ms)	$F_{(2,80)} = 0.87$	$F_{(1,40)} = 1.23$	$F_{(2,80)} = 0.14$
Reaction time variability	0.24 (0.07)	0.23 (0.06)	0.24 (0.06)	0.19 (0.05)	0.19 (0.05)	0.19 (0.05)	$F_{(2,80)} = 0.93$	$F_{(1,40)} = 7.75$**	$F_{(2,80)} = 0.02$

† $p < 0.1$, * $p < 0.05$, ** $p < 0.01$, *** $p < 0.001$

Supplementary Table 2. Means, standard deviations and results of ANOVA for the first, the second and the third session.

	ADHD sham	ADHD anodal	ADHD cathodal	Control sham	Control anodal	Control cathodal	Main effect tDCS	Main effect group	Interaction tDCS x group
Commission errors	14.9% (8.7%)	10.1% (7.0%)	10.3% (8.7%)	11.4% (6.0%)	7.1% (7.7%)	6.3% (7.6%)	$F_{(2,80)}$ = 15.71***	$F_{(1,40)}$ = 2.80	$F_{(2,80)}$ = 0.13
Omission errors	3.6% (5.5%)	5.7% (10.3%)	6.3% (12.8%)	1.6% (2.7%)	1.4% (4.7%)	1.9% (6.6%)	$F_{(2,80)}$ = 1.49	$F_{(1,40)}$ = 2.65	$F_{(2,80)}$ = 1.16
Reaction time	570 ms (42 ms)	562 ms (50 ms)	568 ms (52 ms)	571 ms (92 ms)	538 ms (76 ms)	528 ms (69 ms)	$F_{(2,80)}$ = 6.81**	$F_{(1,40)}$ = 1.23	$F_{(2,80)}$ = 4.88*
Reaction time variability	0.24 (0.06)	0.23 (0.06)	0.24 (0.06)	0.21 (0.04)	0.18 (0.05)	0.18 (0.06)	$F_{(2,80)}$ = 3.88*	$F_{(1,40)}$ = 7.75**	$F_{(2,80)}$ = 3.04†

† $p < 0.1$, * $p < 0.05$, ** $p < 0.01$, *** $p < 0.001$

Supplementary Table 3. Means, standard deviations and results of ANOVA in the first session for sham, anodal and cathodal tDCS.

	ADHD sham	ADHD anodal	ADHD cathodal	Control sham	Control anodal	Control cathodal	Main effect tDCS	Main effect group	Interaction tDCS x group
Commission errors	20.57% (9.19%)	9.82% (7.21%)	14.45% (6.94%)	12.08% (7.25%)	13.21% (6.29%)	8.97% (4.29%)	$F_{(2,36)} = 2.11$	$F_{(1,36)} = 2.66$	$F_{(2,36)} = 2.71$†
Omission errors	5.80% (7.60%)	2.22% (4.60%)	2.79% (3.73%)	1.16% (2.90%)	2.07% (3.21%)	1.47% (2.06%)	$F_{(2,36)} = 0.44$	$F_{(1,36)} = 2.26$	$F_{(2,36)} = 0.98$
Reaction time	585 ms (38 ms)	581 ms (43 ms)	543 ms (36 ms)	557 ms (99 ms)	579 ms (107 ms)	577 ms (81 ms)	$F_{(2,36)} = 0.26$	$F_{(1,36)} = 0.003$	$F_{(2,36)} = 0.64$
Reaction time variability	0.27 (0.07)	0.20 (0.05)	0.23 (0.05)	0.20 (0.02)	0.23 (0.03)	0.21 (0.05)	$F_{(2,36)} = 0.52$	$F_{(1,36)} = 3.13$†	$F_{(2,36)} = 3.47$*

† $p < 0.1$, * $p < 0.05$, ** $p < 0.01$, *** $p < 0.001$

Supplementary Table 4. Commission errors in ADHD patients, separated for session number depending on tDCS condition (mean and standard deviation).

first session	sham:		anodal:		cathodal:	
(n = 7 per group)	20.57% (9.19%)		9.82% (7.21%)		14.45% (6.94%)	
second session	anodal:	cathodal:	sham:	cathodal:	sham:	anodal:
(n = 3 or 4 per group)	10.63% (6.11%)	11.52% (7.06%)	3.28% (1.97%)	12.84% (6.70%)	8.44% (7.57%)	13.40% (8.75%)
third session	cathodal:	anodal:	cathodal:	sham:	anodal:	sham:
(n = 3 or 4 per group)	12.62% (13.07%)	13.02% (15.75%)	4.15% (3.22%)	11.84% (6.54%)	8.56% (5.46%)	10.68% (6.84%)

Supplementary Table 5. Commission errors in control group, separated for session number depending on tDCS condition (mean and standard deviation).

first session	sham:		anodal:		cathodal:	
(n = 7 per group)	12.08% (7.25%)		13.21% (6.29%)		8.97% (4.29%)	
second session	anodal:	cathodal:	sham:	cathodal:	sham:	anodal:
(n = 3 or 4 per group)	9.62% (11.76%)	4.70% (2.60%)	5.13% (2.80%)	10.52% (13.59%)	5.99% (4.30%)	5.27% (1.73%)
third session	cathodal:	anodal:	cathodal:	sham:	anodal:	sham:
(n = 3 or 4 per group)	7.46% (9.02%)	3.56% (1.92%)	5.28% (2.43%)	11.80% (14.20)	4.81% (4.93%)	3.13% (2.85%)
